# Hyaluronan synthase mediates dye translocation across liposomal membranes

**DOI:** 10.1186/1471-2091-13-2

**Published:** 2012-01-25

**Authors:** Andria P Medina, Jialing Lin, Paul H Weigel

**Affiliations:** 1 Department of Biochemistry & Molecular Biology, The University of Oklahoma Health Sciences Center (940 S. L. Young Blvd), Oklahoma City, OK 73104, USA; 2 The Peggy and Charles Stephenson Oklahoma Cancer Center, The University of Oklahoma Health Sciences Center (940 S. L. Young Blvd), Oklahoma City, OK 73104, USA; 3 The Oklahoma Center for Medical Glycobiology, The University of Oklahoma Health Sciences Center (940 S. L. Young Blvd), Oklahoma City, OK 73104, USA

## Abstract

**Background:**

Hyaluronan (HA) is made at the plasma membrane and secreted into the extracellular medium or matrix by phospolipid-dependent hyaluronan synthase (HAS), which is active as a monomer. Since the mechanism by which HA is translocated across membranes is still unresolved, we assessed the presence of an intraprotein pore within HAS by adding purified *Streptococcus equisimilis *HAS (SeHAS) to liposomes preloaded with the fluorophore Cascade Blue (CB).

**Results:**

CB translocation (efflux) was not observed with mock-purified material from empty vector control *E. coli *membranes, but was induced by SeHAS, purified from membranes, in a time- and dose-dependent manner. CB efflux was eliminated or greatly reduced when purified SeHAS was first treated under conditions that inhibit enzyme activity: heating, oxidization or cysteine modification with N-ethylmaleimide. Reduced CB efflux also occurred with SeHAS K48E or K48F mutants, in which alteration of K48 within membrane domain 2 causes decreased activity and HA product size. The above results used liposomes containing bovine cardiolipin (BCL). An earlier study testing many synthetic lipids found that the best activating lipid for SeHAS is tetraoleoyl cardiolipin (TO-CL) and that, in contrast, tetramyristoyl cardiolipin (TM-CL) is an inactivating lipid (Weigel et al, J. *Biol. Chem*. **281**, 36542, 2006). Consistent with the effects of these CL species on SeHAS activity, CB efflux was more than 2-fold greater in liposomes made with TO-CL compared to TM-CL.

**Conclusions:**

The results indicate the presence of an intraprotein pore in HAS and support a model in which HA is translocated to the exterior by HAS itself.

## Background

Hyaluronic acid (hyaluronan; HA) is a linear unbranched polysaccharide composed of repeating disaccharide units of *N*-acetyl-glucosamine and D-glucuronic acid. In vertebrates and streptococcal species, HA is synthesized by Class I HA synthases (HAS), lipid-dependent integral membrane proteins with eight membrane domains (MDs) in eukaryotes or six MDs in species such as *S. pyogenes *and *S. equisimilis *[1,2]. Mammals express three HAS isozymes encoded by distinct genes designated HAS1, HAS2 and HAS3. HA is an essential glycosaminoglycan in vertebrate extracellular matrices [3], where it helps maintain the physical structure and integrity of tissues, in particular cartilage. HA is also a major constituent of skin, vitreous humor in the eye, synovial fluid in joints, and the cumulus cell matrix that surrounds oocytes prior to ovulation. In tissue matrices or vitreous, HA ranges in molecular mass up to 10 MDa (25, 000 disaccharide units) and occupies a very large volume in physiological fluids. In addition to its physical functions, it is now clear that HA has multiple different size-dependent functions and is capable of stimulating intracellular signal transduction to alter gene expression and cell behavior in angiogenesis, inflammatory diseases, tumorigenesis and metastasis [4-8].

Two mechanisms have been suggested for how HA is transferred across the cell membrane to the exterior. The first [9] is that HAS itself, which is active as a monomer in complex with multiple lipids molecules [10], mediates HA translocation via a pore within the multiple MDs of the protein. The second is that a growing HA chain in the cytoplasm binds to an ABC transporter, which then transfers the chain across the membrane [11,12]. This suggestion arose when mutation of a gene near the *S. pyogenes has *operon reduced HA synthesis and secretion by about 65%, although SpHAS activity in lysed cells was unaffected. The mutated gene had sequence similarity to bacterial ABC transporter genes. In eukaryotic cells, the current proposal is that both the multiple drug resistance protein-5 (MRP5; ABCC5) and the cystic fibrosis conductance regulator are ABC transporters that would bind and translocate intracellular HA to the exterior in an ATP-dependent manner [13,14].

The HAS Pore model is based on three aspects of HAS structure and function that are very novel for a glycosyltransferase: the lipid-dependence, the large number of MDs, and the processive mechanism of HA synthesis. Very few glycosyltransferases are multiple membrane spanning proteins and dependent on lipid for activity. HA·HAS complexes remain associated for 2-4 hr until the completed MDa HA chains are released [15]. Additional support for the Pore model is that insertion of a HAS gene into cells that do not otherwise make HA can confer the ability to synthesize and secrete HA in prokaryotes [16,17] or eukaryotes such as drosophila [18]. These results are readily explained by the ability of HAS alone to both assemble and translocate HA. The ABC transporter model explains these latter results by proposing that the transporters in cells that do not make HA are promiscuous enough to transport not only their normal substrates, but also the large HA polysaccharide made when a HAS gene is introduced. A further distinction is that in contrast to the ABC transporter model, the HAS Pore model with coupled HA synthesis and translocation does not require ATP.

In this study, we tested a central prediction of the Pore model, that HAS contains an intraprotein pore, by assessing the ability of purified SeHAS to mediate efflux (*i.e*. translocation) of the small fluorescent dye Cascade Blue (CB, 538.4 Da) from the inside of pre-loaded liposomes to the external medium. The results indicate that SeHAS can mediate CB efflux when added to liposomes and that this pore-like activity is eliminated or impaired by a range of treatments or conditions that inhibit the synthase activity of SeHAS.

## Results

### CB Efflux occurs after SeHAS addition to liposomes

**Figure 1:**
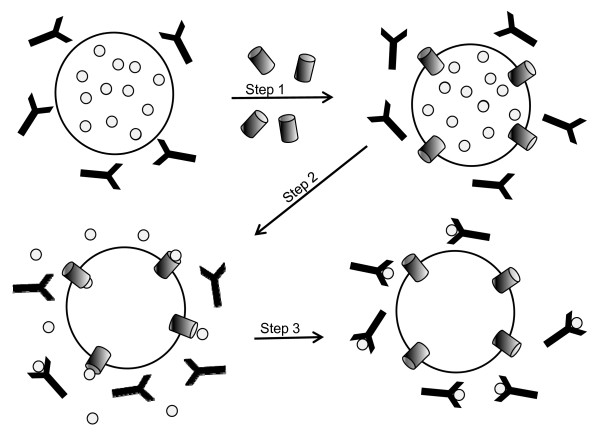
**Experimental model for monitoring HAS-mediated FL quenching in liposomes**. Step 1 in the scheme depicts the addition of purified SeHAS (cylinders) to extrusion liposomes (large circles) containing free CB (small circles). In step 2, HAS molecules insert into the liposome bilayer, allowing the small CB dye to translocate (efflux) through the HAS pores to the exterior if an intraprotein pore is present. In experiments with continuous FL monitoring in the presence of external anti-CB Ab (black Y), CB that effluxes from the lumen is bound by Ab resulting in FL quenching (step 3).

**Figure 2:**
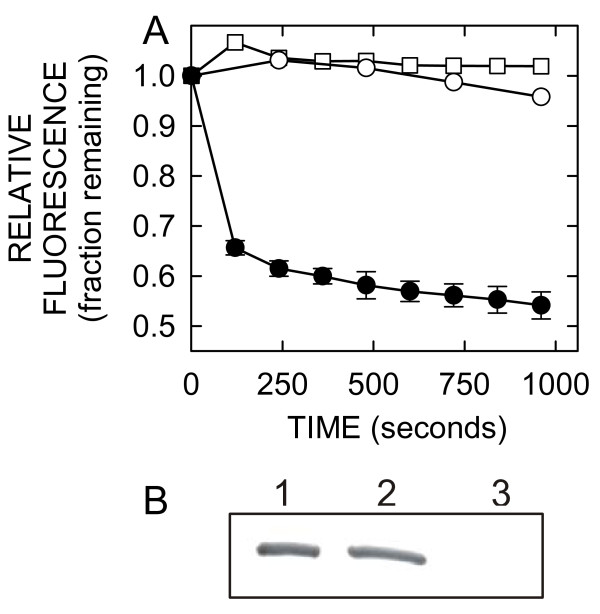
**Incorporation of SeHAS into extrusion liposomes induces CB quenching**. A. Extrusion liposomes loaded with CB were prepared, purified and stored at 4°C under argon as described in Methods. Liposomes were suspended in 0.25 ml 0.1 M NaCl, 51 mM sodium phosphate, 3.8 mM Na citrate, pH 7.4 (~50 μM PL) with 10 μg/ml anti-CB Ab and equilibrated at 30°C in cuvettes in the fluorometer. FL was monitored at 433 nm for 5 min to obtain a stable base-line (time-zero), and then SeHAS (15 μl; purified with no DDM or glycerol in the elution buffer) was added to 200 nM (black circles; mean ± SE, n = 3) or the same volume of mock-purified empty-vector membranes (white circles) or elution buffer (white squares) was added. Samples were mixed well and FL monitoring was continued. Decreasing FL (ongoing quenching) was observed after SeHAS addition, but not after addition of elution buffer or mock-purified material from empty-vector membranes. B. Purified SeHAS was added to extrusion liposomes as in A and after 30 min the suspension was centrifuged, the supernatant was saved, and the pellet was resuspended and centrifuged. The final pellet (lane 2) was resuspended to the same volume as the original supernatant (lane 3), trichloroacetic acid was added (to 10%; w/v) to both samples and precipitated protein was centrifuged, redissolved in Laemmli buffer and analyzed by SDS-PAGE and Western blotting with anti-SeHAS Ab. Lane 1 is a sample of purified SeHAS precipitated and redissolved in parallel as a control.

When CB dye is released from liposomes and bound by anti-CB antibody (Ab) in the external phase (Figure 1), its fluorescence (FL) is quenched. Luminal CB does not readily leak through the liposomoal membrane, and hence, is protected from the quenching. However, CB release can occur in several ways, including detergent mediated lysis, loss of liposome integrity (*e.g*. due to aging-induced lipid oxidation and degradation), or by passage through pores formed by insertion of proteins into liposomal membranes [19,20]. For these experiments, SeHAS was purified using a protocol with reduced n-dodecyl-β-D-maltoside (DDM) and glycerol concentrations to minimize carryover of any detergent. Prior to protein addition or in the absence of addition, a stable FL baseline was observed, indicating that no CB dye is released spontaneously from liposomes at 30°C. After SeHAS addition, there was a time-dependent decrease in FL (an increase in quenching) indicating an ongoing efflux of CB (Figure 2A). In contrast, there was no quenching when the same buffer without SeHAS or when mock-purification material from empty-vector membranes was added. The latter control indicates that CB efflux from liposomes was not caused by residual DDM or other membrane contaminants. Essentially all of the added SeHAS was incorporated into the liposomes under these conditions (Figure 2B). Additionally, SeHAS did not cause dye release and quenching when added to liposomes containing a larger (3 kDa) dextran-CB conjugate (not shown).

### CB efflux from liposomes is dependent on SeHAS concentration

**Figure 3:**
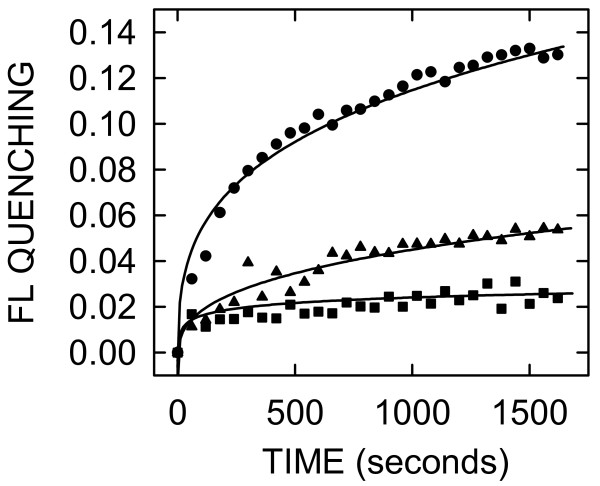
**SeHAS-dependent quenching in extrusion liposomes is concentration dependent**. Different amounts of purified SeHAS, in the same volume of Elution Buffer, were added to CB-containing liposomes at 30°C to final concentrations of 50 (black squares), 100 (black triangles), or 200 (black circles) nM and FL changes were recorded as in Fig 2. In this and the following Figs, FL quenching (1.0 - relative FL) is graphed, rather than relative FL. After 1800 seconds, quenching was 3%, 6%, and 14% in response to addition of 50, 100 and 200 nM SeHAS, respectively.

If CB efflux is mediated by SeHAS insertion into liposomes, this effect should be dose dependent. When this was assessed using different amounts of purified SeHAS, the resulting CB efflux was dependent on SeHAS concentration (Figure 3). After adding SeHAS to 50, 100, or 200 nM, the quenching after 30 min was 3%, 6% and 14%, respectively. Thus, essentially a linear response in CB efflux was observed with increasing SeHAS concentration, consistent with the interpretation that CB efflux is mediated by, and dependent on, SeHAS.

### SeHAS-mediated CB efflux is greater from liposomes made with an activating compared to an inactivating lipid

If CB efflux is mediated by native active SeHAS, then this response should be increased by conditions that stimulate enzyme activity and decreased by treatments that inhibit or inactivate enzyme activity. SeHAS and other Class I HAS enzymes are lipid-dependent [1,9,21]. For SeHAS, we found that tetraoleoyl cardiolipin (TO-CL), containing C18:1 oleoyl groups (with one double bond), is the best activating phospholipid, whereas tetramyrstoyl cardiolipin (TM-CL) with saturated C14:0 myristyl groups actually inhibits SeHAS activity [22]. Earlier radiation inactivation studies found that active native and recombinant streptococcal synthases are a complex of one HAS monomer and ~16 cardiolipin molecules [10]. The liposomes used here were made with 5% bovine cardiolipin (BCL), which contains several CL species including TO-CL and is an activating lipid for SeHAS [21].

**Figure 4:**
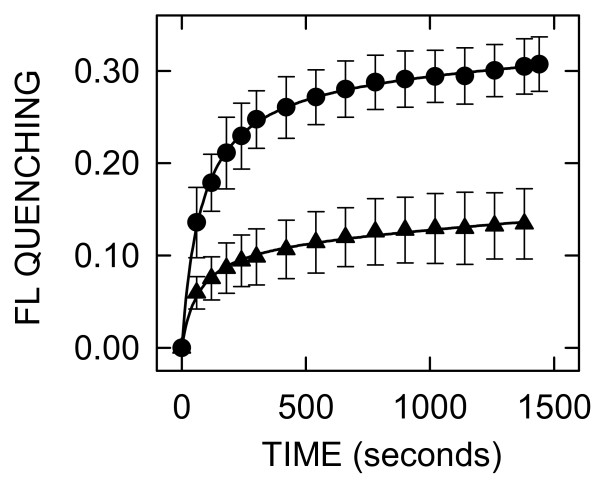
**SeHAS mediates greater CB release from extrusion liposomes containing activating TO-CL compared to inhibitory TM-CL**. Purified SeHAS was added to liposomes containing (mol%) 70% PE, 25% PG and either 5% TO-CL (black circles) or TM-CL (black triangles). Subsequent release of CB and FL quenching by anti-CB Ab was monitored as described in Fig 2. Values are the mean ± SE (n = 3). Note that the FL quenching scale is greater than in other Figs.

To determine if CB efflux mediated by SeHAS is influenced by the CL species present, we tested liposomes made with an *E. coli*-based lipid composition of phosphatidyglycerol (PG) and phosphatidyethanolamine (PE) and either 5% TO-CL or TM-CL, in place of BCL. The maximal quenching induced by SeHAS addition to liposomes made with TO-CL was more than 2-fold greater than in liposomes made with TM-CL (Figure 4). Interestingly, quenching using TM-CL liposomes was ~20% less compared to the standard liposomes made with BCL, but quenching in liposomes made with TO-CL was ~50% greater.

### Fluorescence quenching does not occur after addition of heat-inactivated, oxidized or Cys-modified SeHAS

**Figure 5:**
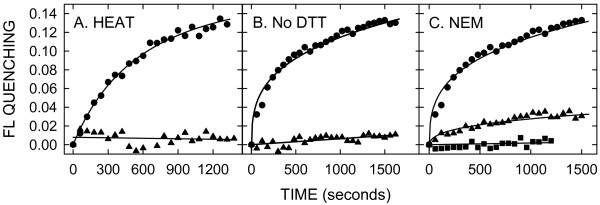
**SeHAS inactivated by heat, oxidation or NEM modification does not mediate CB release from extrusion liposomes**. A. Heat inactivation. CB-containing liposomes were treated at 30°C as in Fig 2 except that before addition to liposomes, purified SeHAS was either treated at 47°C for 5 min to inactivate the enzyme (black triangles) or left untreated on ice (black circles). FL quenching was monitored as in Fig 2. B. Oxidative inactivation. Purified oxidized SeHAS was prepared by omitting DTT in the elution buffer. Oxidized SeHAS was then either left oxidized (black triangles) or was reduced by treatment with 1 mM DTT for 1 hr at 4°C (black circles) and then added to extrusion liposomes and FL quenching monitored as in Fig 2. The response with reduced and rescued SeHAS is essentially identical to the untreated enzyme in A. C. NEM modification. Purified SeHAS was untreated (black circles) or treated (black triangles) for 15 min at 4°C with 6 mM NEM, which inhibits SeHAS activity (24); DTT (12 mM) was then added to react with the remaining NEM. SeHAS was added to CB-liposomes to a final concentration of 200 nM and FL changes were monitored as in Fig 2. CB-liposomes without SeHAS were also incubated in the same way with NEM and DTT as a control (black squares) to assess possible leakage.

To determine if the observed CB efflux required active native SeHAS, we inactivated the enzyme by heating for 5 min at 47°C [21]. There was no quenching of CB FL when this inactive SeHAS was added to liposomes (Figure 5A), indicating that native enzyme is required. It has been known for > 50 years that HA synthesis does not occur if the enzyme is oxidized; thus, a reducing agent is required to maintain enzyme activity [23]. To assess the ability of oxidized SeHAS to mediate CB efflux, we omitted DTT during the standard elution step, which resulted in inactive enzyme (not shown) that was unable to mediate CB efflux from liposomes (Figure 5B). Reducing the oxidized SeHAS by adding back DTT recovered both enzymatic activity and the ability to mediate CB efflux. Although Cys residues in SeHAS or SpHAS are not required for enzyme activity, their modification by NEM or other sulfhydryl reagents drastically reduces activity [24-26]. Adding SeHAS pretreated with NEM to liposomes resulted in ~75% less quenching compared to unmodified enzyme, whereas addition of NEM alone did not quench CB FL (Figure 5C). Thus, three different treatments that inhibit HAS activity also decreased the ability of SeHAS to mediate CB efflux.

### CB efflux from liposomes is reduced in SeHAS MD mutants

**Figure 6:**
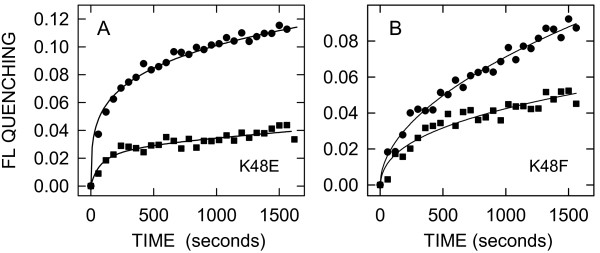
**K48E and K48F mutants mediate less CB release from liposomes than WT SeHAS**. Purified WT (black circles) and K48E (Panel A, black squares) or K48F (Panel B, black squares) SeHAS were added to CB-containing liposomes and FL quenching was monitored at 30°C for the indicated times as in Fig. 2.

We reported previously that two charged residues within MD2 (Lys^48^) and MD4 (Glu^328^) appear to act together (*e.g*. as an ion pair) to stabilize the protein and allow synthesis of very large HA [27]. These two MD residues are within the proposed intraprotein pore of HAS. The *V_max _*of SeHAS(K48E), a charge-switch mutant, is reduced by 93% and it makes HA that is 81% smaller compared to WT SeHAS. CB quenching after addition of SeHAS(K48E) to liposomes was reduced by 66% compared to WT (Figure 6A). We also tested a K48F variant because the replacement residue has a bulky side group and this mutant makes HA that is ~77% smaller than WT. FL quenching was reduced by ~45% compared to addition of WT SeHAS (Figure 6B). These results indicate that CB efflux mediated by SeHAS is inhibited by mutations in the putative pore region that decrease both enzyme activity and HA product size.

## Discussion

The present results demonstrate that purified HAS reconstituted into liposomes mediates luminal CB efflux in a manner consistent with the presence of a HAS pore. HAS-mediated dye efflux was time-dependent and HAS concentration-dependent, and efflux did not occur or was greatly impaired if the enzyme was first inactivated by heat treatment or Cys -SH group modification by either oxidation or reaction with NEM. These results indicate that active native enzyme is required for efflux. HAS enzyme function is greatly affected by whether sufficient activating phospholipid is present. We previously found that purified SeHAS retains enough tightly bound endogenous lipid to retain low activity (and not be denatured), but the enzyme can be activated ≥ 10-fold by addition of TO-CL, containing C18:1 oleic acid chains; the best activating lipid identified so far [22]. In contrast, TM-CL, containing C14:0 saturated fatty acids, does not activate SeHAS. The finding that HAS-mediated dye efflux was greater in liposomes with TO-CL compared to BCL and greater with BCL versus TM-CL is consistent with the presence of a pore whose size is dependent on the associated CL species.

An intraprotein pore in HAS-CL complexes may be smaller or distorted (thus slowing the rate of efflux) in the presence of the "bad" TM-CL species. Unlike other phosholipids, CL (also called diphosphatidylglycerol) has a "double" head group and four fatty acyl chains. Consequently, whereas other lipids occupy roughly cylindrical volumes, CL is shaped more like a cone with the narrower head group at the top. This structure imparts a natural tendency for CL to create or to localize to areas of negative (*i.e*. concave) membrane curvature [28]. We conclude that HAS could be activated by TO-CL, which favors negative curvature in the inner leaflet, because it would decrease lateral pressure on the protein and thus open up the cytoplasmic active sites and intraHAS pore [29]. In contrast, HAS inhibition by TM-CL, whose shorter unkinked acyl chains favor a more cylindrical shape and more positive membrane curvature, could occur because it increases lateral pressure on the protein and would narrow the pore.

Since cloning and characterizing the first HAS [30], we have been interested in understanding how HAS regulates the size of HA chains it assembles and how HA·HAS complexes can stay together for hours without dissociating during continuous polymerization of a growing chain at the reducing end (unlike the vast majority of glycosyltransferases that act at the nonreducing end). The characteristics of the HAS protein itself and how it functions led us to propose the Pore model for HA translocation through and by a HAS-lipid complex [1,9,31]. For example, the lipid-dependence and 6-8 MDs of membrane-bound HAS enzymes are characteristics common to pore forming proteins [19,32-35]. Additionally, the substrate binding sites are on the cytoplasmic side of the protein close to the cytoplasm-membrane interface [2,26] and the enzyme is highly processive [15,36].

Processive biosynthesis requires that after each catalytic cycle the HA-UDP product must transiently dissociate, without being released to diffusion, move relative to the enzyme active sites, and then rebind to HAS for the next cycle of sugar additions. As with other polymerases, the binding interaction between enzyme and polymer substrate cannot be of such high affinity that the rate of release, and thus rebinding of polymer, is too slow. Many RNA and DNA enzymes (*e.g*. polymerases and topoisomerases) have evolved solutions to this dilemma by creating topological or spatial constraints so that enzyme and product cannot dissociate. Molecular tethering strategies include formation of either multi-protein complexes that surround and encase the polymeric substrate or an intra-enzyme channel or pore to achieve a similar entrapment of the polymer.

This processivity of HA·HAS complexes is most consistent with the Pore model, which provides a topological mechanism to prevent dissociation during HA elongation. Since the 6-8 MDs of HAS enzymes are associated with multiple lipid molecules, it seems most likely that they utilize an intraHAS pore or deep cleft within the membrane that simultaneously serves to topologically constrain and also to provide a translocation mechanism to move the growing chain to the cell exterior. When dissociation does occur, HA release appears to terminate further elongation, perhaps due to loss of UDP at the reducing end - a chain terminating event. Even high affinity interactions (*e.g*. between receptors and ligands) typically have nM K_d _values and measurable off-rates. The extremely slow, essentially immeasurable, off-rate for HA·HAS complex dissociation is inconsistent with enzyme reactions that use soluble substrates, which typically have K_m _values in the μM range, as proposed in the ABC transport model.

We previously identified Lys^48 ^and Glu^327 ^in MD2 and MD4, respectively, as important residues for the ability of SeHAS to produce large MDa HA [27]; the K48E and K48F mutants make much smaller HA than WT. The interpretation that these two residues are within the proposed HAS pore and may interact with each other or with the growing HA chain during translocation is supported by the present findings that CB efflux mediated by both proteins is decreased compared to WT.

Since the present evidence supports the presence of an intraHAS pore through which HA could be translocated across membranes, it is reasonable to consider alternative interpretations that could account for reduced HA synthesis and secretion by inhibition of ABC transporters, such as MRP5. Two other explanations for decreased HA synthesis and secretion after inhibition or knockdown of MRP5 and other ABC transporters are related to the ability of these proteins to transport nucleotides (*e.g*. cGMP, cAMP) as well as nucleotide-based drugs such as 5'-Fluoro-UMP, a metabolite of the anticancer drug 5'-Fluorouracil [37]. The first possibility is that HAS inhibition could be caused by perturbation of the normal cellular metabolism of uracil-containing nucleotides, resulting in altered UDP-sugar concentrations or ability of precursor pathways to sustain the metabolic flux needed to make large amounts of HA. In eukaryotes, UDP-sugars are synthesized in the cytoplasm and levels are controlled by several mechanisms, including the Golgi anti-port system in which a UDP-sugar is transported in, while UMP is transported out. UMP deficiency causes the metabolic disorder orotic aciduria, but excess UMP (which leads to excess UDP) would also have a detrimental effect on HA synthesis. UDP is a potent substrate inhibitor of all Class I HASs [1] and, in fact, can be used to quench HA synthase activity [15]. Thus, deficiency or inhibition of MRP5 could lead to accumulation of cytoplasmic UDP and inhibition of HAS, an indirect result of MRP5 function unrelated to HA translocation. Both of the above possible indirect effects on HAS activity would be reversed by lysing cells (*i.e*. dilution and mass action effects) and performing assays with UDP-sugars added *in vitro*. Although these and other alternate explanations remain to be tested, Thomas and Brown recently found that ABC transporters are not involved in HA export by cancer cells [38].

## Conclusions

We conclude that HAS contains an intraprotein pore capable of allowing small molecules such as Cascade Blue (538.4 Da) to pass across liposomal membranes. Such a pore within HAS provides both an explanation for the extreme processive behaviour of HAS·HA complexes, which remain associated for hours during biosynthesis of MDa HA chains, and a mechanism for the translocation of HA across cell membranes to the cell exterior that is consistent with the enzyme's structure and lipid dependence.

## Methods

### Materials and Buffers

Media components were from Difco (Fisher Scientific). Plasmid pKK223-3 was from GE Healthcare and *E. coli*, SURE™ cells were from Stratagene (La Jolla, CA). Ni^2+^-nitrilotriacetic acid (Ni-NTA) resin was from Qiagen Inc (Hilden, Germany). CB (8-methoxypyrene-1, 3, 6-trisulfonic acid, trisodium salt, 538.4 Da) and anti-CB quenching antibody Ab were from Invitrogen-Molecular Probes. Synthetic TO-CL and TM-CL were from Avanti Polar Lipids (Alabaster, Alabama). BCL, *E. coli *PE, and chicken egg PG were from either Avanti or Sigma-Aldrich. Other reagents were from Sigma-Aldrich (St. Louis, MO). Liposome Buffer contains 100 mM NaCl, 51 mM Na_2_HPO_4_, 3.8 mM citric acid, pH 7.4. Extraction Buffer contains 10 mM DDM, 50 mM sodium and potassium phosphate, pH 7.0, 150 mM NaCl, 10 mM MgCl_2_, 1.0 mM β-mercaptoethanol, 20% glycerol, 0.5 μg/ml leupeptin, 0.7 μg/ml pepstatin, and 46 μg/ml phenylmethylsulfonyl fluoride. Wash and Elution Buffers contain the same components except that in Wash Buffer glycerol is 10% and DDM is 1 mM, and Elution Buffer does not contain either glycerol or DDM.

### Cell growth and membrane purification

The HAS open reading frame from *S. equisimilis*, with a C-terminal His_6 _fusion, was inserted into the pKK223-3 vector and cloned into *E. coli *SURE-2 cells [17], and cell growth, lysozyme/EDTA treatment, and membrane preparation in the presence of protease inhibitors were performed as described previously [9,21]. Final membrane pellets were stored at -80°C.

### Enzyme extraction and purification

In order to minimize the residual detergent associated with purified SeHAS to be used with liposomes, the Extraction, Wash and Elution buffers used in previous purification schemes were modified, as noted above, to reduce or eliminate glycerol and DDM. Thawed membrane pellets were solubilized in 10 ml of Extraction Buffer for 2 h at 4°C with gentle mixing in a Micromixer E-36 (Taitec), followed by centrifugation at 100, 000 × g for 1 h at 4°C to sediment insoluble components. Imidazole was added to the supernatant to a final concentration of 30 mM to minimize nonspecific binding of *E. coli *proteins and the extract was incubated for 30 min at 4°C with constant mixing with Ni^2+^-depleted NTA resin (0.3 ml in a mini-spin column; Bio-Rad) equilibrated with Extraction Buffer without MgCl_2_. The enzyme extract was removed and then incubated with 0.3 ml Ni^2+^-NTA resin for 2 h at 4°C with constant mixing. After allowing the resin to pack, unbound proteins were allowed to flow through, the resin was washed with 10 volumes of Wash Buffer to remove contaminants, and bound SeHAS was recovered with 0.3 ml Elution Buffer. SeHAS protein was determined with the Coomassie protein assay reagent (Pierce) using bovine serum albumin as the standard, and purity was assessed by SDS-PAGE; preparations were typically > 98% pure based on densitometric analysis of Coomassie-stained gels.

### Extrusion liposomes

Liposomes containing CB were prepared as described by Szoka et al [39,40]. Since recombinant SeHAS in *E. coli *membranes has been well characterized and is very active, we prepared liposomes (unless noted otherwise in particular experiments) using a phospholipid mixture based on *E. coli *membranes: 75% PG, 20% PE and 5% CL (in mole percents). To facilitate binding between liposomes and the C-terminal His_6 _of SeHAS [40], we also added 1% of a NTA-Ni^2+ ^PG analogue (Avanti): 1, 2-dioleoyl-*sn*-glycero-3-[(N-(5-amino-1-carboxypentyl)iminodiacetic acid)-succinyl] (nickel salt). Lipids in chloroform or ethanol were mixed and dried under a partial vacuum for 3 h at 37°C in a N_2 _atmosphere. To the dried lipid mixture was added 0.55 ml of Liposome Buffer and 30 μM CB [19]. The mixture was incubated at 37°C for 30 min, and then resuspended by vortex mixing for 5 min. The lipid mixture was frozen in liquid N_2 _and thawed in a 37°C water bath five times to reduce the content of multilamellar liposomes and to increase liposome luminal volume, and then passed 21 times through a 0.2 μm pore size polycarbonate filter at room temperature using a Lipofast extruder (Avestin Inc, Ottawa, Canada) to make unilamellar liposomes [39]. Liposomes were stored at 4°C until use, and then external CB was removed from liposomes (containing internal CB) by either centrifugation or size exclusion chromatography (SEC) over Sephadex G-25.

### Monitoring CB release from Extrusion liposomes

CB-loaded liposomes (50 μM phospholipid) were equilibrated in 0.250 ml Liposome Buffer containing 6-10 μg/ml anti-CB Ab at 30°C in a cuvette in an SLM-8100 spectrofluorometer [19]. Gentle mixing was achieved with a flea magnet and magnetic stirrer. FL intensity was measured (excitation at 405 nm and emission at 433 nm) for at least 5 min until a steady baseline was achieved. CB release from liposomes, upon addition of SeHAS or other agents, was monitored continuously by measuring CB FL in the presence of external anti-CB Ab that quenches FL. Purified SeHAS (0.83 μM) was then added to the cuvette to a concentration of 50 nM, the contents mixed, and continuous FL monitoring resumed 20 s after SeHAS addition (this time point is designated as t_o _or 0 sec in Figures 2, 3, 4, 5, and 6). At the end of each kinetic run, Triton-X100 was added to a final concentration of 0.1% (v/v) to lyse liposomes, completely release CB, and obtain values for maximum (100%) quenching by the anti-CB Ab. Relative FL is calculated as [(F_t_-F_T_)/(Ft_0_-F_T_)] where: Ft_0 _= FL at time-zero with anti-CB Ab present, but before SeHAS addition to liposomes; F_t _= FL at a given time t, and F_T _is the final FL 5 min after adding Triton-X100. FL quenching (Q) is calculated as: Q = (Ft_0 _- F_t_)/(Ft_0 _- F_T_). Thus, at any given time Q represents the efflux and quenching of CB as a fraction of the total FL quenching of CB after Triton X-100 addition. Q ranges from a value of 0 at t_0 _to 1.0 after Triton addition.

## Abbreviations

Ab: antibody; BCL: bovine cardiolipin; CB: Cascade Blue; DDM: n-dodecyl-β-D-maltoside; FL: fluorescence; HA: hyaluronic acid: hyaluronate: hyaluronan; HAS: HA synthase; MD: membrane domain; MRP5: multidrug resistant protein 5; NEM: N-ethylmaleimide; PBS: phosphate buffered saline; PE: phospatidyl ethanolamine; PG: phosphatidyl glycerol; PL: phospholipid; SeHAS: *Streptococcus equisimilis *HAS; SpHAS: *Streptococcus pyogenes *HAS; TM-CL: tetramyristoyl cardiolipin TO-CL: tetraoleoyl cardiolipin.

## Authors' contributions

APM performed all the experiments and helped interpret results and draft the manuscript. JL served as an expert consultant regarding liposomes, helped plan liposome-based studies, provided instrument and software training and helped draft the manuscript. PHW conceived of and planned the approach, and helped design experiments, interpret results, and draft the manuscript. All authors have read and approved the final manuscript.
